# Factors Influencing Procurement Officers’ Preference for PPP Procurement Model: An Empirical Analysis of China

**DOI:** 10.3389/fpsyg.2022.832617

**Published:** 2022-05-04

**Authors:** Fuguo Cao, Cong Wang

**Affiliations:** ^1^School of Law, Central University of Finance and Economics, Beijing, China; ^2^School of Finance and Taxation, Central University of Finance and Economics, Beijing, China

**Keywords:** tendering, negotiation, PPP, procurement model, selection

## Abstract

The selection of the procurement model, which is a process of discretion exercised by procurement officials, is crucial to the Public-private Partnership (PPP) procurement performance. From theoretical analysis and international practice, we could find that the negotiation method is more suitable for complex PPP projects, while the tendering method is widely used in China’s PPP procurement. To analyze the reasons for the phenomenon, we used the logit regression model to examine the influence of regulatory competition, risk aversion preference, and tacit interaction on the procurement model selection based on the data of 8,926 PPP projects from 2009 to 2021 in China. The results indicate that regulatory competition leads to confusion in procurement model selection, while risk aversion preference and tacit interaction significantly promote the application of tendering. Moreover, the heterogeneity analysis of regions and return models prove that provincial capitals and municipalities are more susceptible to regulatory competition, risk aversion preference, and tacit interaction than ordinary cities. Compared with user-pay projects, government-pay projects and viability gap funding projects are more susceptible to regulatory competition and risk aversion preference, and less affected by tacit interaction. Therefore, to optimize the procurement model selection, policymakers should improve procurement policies to reduce the adverse effects of regulatory competition, risk aversion preference, and tacit interactions.

## Introduction

The application, selection, and design of procurement models are the core elements of government procurement systems (Cao, 2000). The appropriate procurement model contributes to selecting the best private sector (Wang et al., 2019) and the best design solution (Herweg and Schmidt, 2017). Moreover, it reduces the risk of project failure (Estache et al., 2009). Thus, it is an important aspect of achieving procurement performance. Procurement law provides a system of procurement models for officials to select the appropriate models in different situations. Although the expression of these models varies slightly from one procurement system to another^1^, procurement models also typically include tendering method and negotiation method. To be specific, the tendering method is used for projects in that the procurement specifications are complete, clear, and objective^2^, while the negotiation method is suitable for projects that are technically complex or of a specific nature, and detailed specifications cannot be determined^3^. Therefore, the difference between the two is whether purchasers can express specifications completely, clearly, and objectively. If it is, the tendering is applied and there is no need for communication between the purchaser and the supplier. If not, the negotiation is applied and the communications between the purchaser and the supplier are required to determine the specifications.

In traditional procurement, the tendering method is considered to be the most efficient way to achieve value for money, avoid favoritism, and ensure a transparent and competitive process (Chong et al., 2013). Thus, it was established as the preferred method by the government procurement law. Nonetheless, the limitations of the tendering method are evident. It stifles communication between purchasers and suppliers and discourages purchasers from utilizing the expertise of contractors when they design projects. In addition, it is less efficient when the project is complex, the contract design is incomplete and bidders are fewer (Bajari et al., 2009). Moreover, ignoring the characteristics of the project and being in favor of the tendering method will lead to expensive renegotiations (Chong et al., 2014). Furthermore, it is difficult for officials to evaluate the project in complex procurement for lacking information about potential design improvements (Herweg and Schmidt, 2017). Instead, the negotiation method has flexibility and advantages, especially in the procurement of complex projects. It helps buyers and suppliers exchange information early in the process and spend more time discussing the project and eliminating possible defects (Victor, 1977). This not only reduces the likelihood of renegotiation (Estache et al., 2009; Nunzia et al., 2016), but also allows the necessary flexibility in the procurement of complex projects (Bajari et al., 2009). Meanwhile, compared with the tendering method, the negotiation method can effectively motivate suppliers to improve their design and be more likely to achieve project optimization (Herweg and Schmidt, 2017). In addition, procurement practices also prefer the negotiation method in complex projects. For example, public procurement practices in Italy and Northern California have validated the impact of complexity on the selection of procurement models (Bajari et al., 2009; Baldi et al., 2016). The more complex the project is, the more likely it is that the negotiation method will be chosen. Given the above, it is rational to choose the negotiation method in projects with high complexity.

Typically, PPP projects are more complex than traditional procurement projects (Carbonara et al., 2016; Zhen and Cao, 2018). Therefore, the negotiation method is a more appropriate procurement model. In addition, negotiation is the recommended procurement model by *UNCITRAL Model Law on Public-Private Partnerships*^4^. Furthermore, many countries tend to choose negotiation methods in PPP procurement, such as the United Kingdom, Ireland, Portugal, Netherlands, France, and Italy (Soliño and Gago de Santos, 2016). Consequently, the negotiation method is the preferred method in PPP procurement.

**FIGURE 1:**
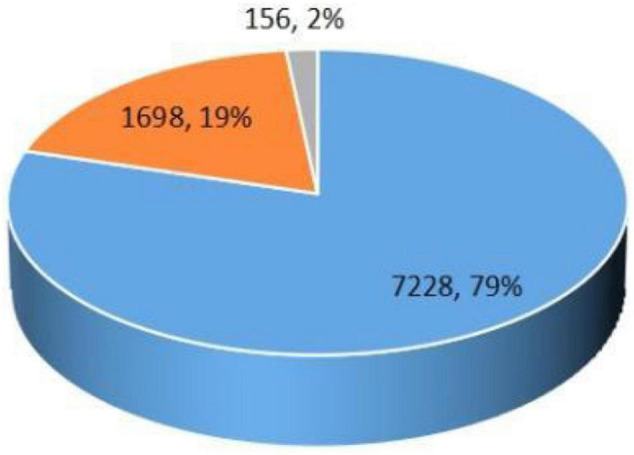
Distribution of procurement model selection.

However, observing the PPP procurement practice in China, we find that the tendering method is applied in a much larger proportion than the negotiation method. According to statistics, from January 1, 2019 to March 21, 2021, there are a total of 9,082 PPP projects in the procurement and execution stages. The number of PPP projects that adopted the tendering method was 7,228, accounting for 79%. Among them, open tendering was used for 7,182 projects, and invited tendering was 46. The number of PPP projects that adopted the negotiation method was 1,698, accounting for only 19%. Among them, competitive consultation was used for 1,604 projects and competitive negotiation was 94^5^ (see Figure 1). Compared with theories, legal designs, and practices in other jurisdictions, this stark contrast deserves to be studied and interpreted. Therefore, we propose the following research question: What factors contribute to the preference for tendering in the procurement of PPP projects in China?

Currently, some scholars have studied the influencing factors of procurement model selection. One of the factors that have received a lot of attention is complexity. For example, Bajari et al. (2009) examined the impact of complexity on the selection of procurement model using a sample of procurement contracts in the construction sector in Northern California from 1995 to 2000. They confirmed that more complex projects were more likely to be procured through the negotiation methods. Carbonara et al. (2016) constructed a conceptual model of PPP procurement decisions based on the transaction cost minimization theory and studied the influencing factors of procurement model selection of the legal framework of the EU. They reported that negotiation procedures and competitive dialog are preferred when project size increases, project complexity increases, and operational complexity is higher than construction complexity. Baldi et al. (2016) explored the influence of project complexity on procurement model selection by analyzing about 11, 400 samples of public works procurement contracts solicited by Italian municipalities from 2009 to 2012. They revealed that the more complex the project is, the more likely it is to use the negotiation method. However, Chong et al. (2014) studied the impact of project complexity on the selection of procurement model on statistical analysis using a sample of public procurement contracts in the construction sector in France between 2005 and 2007. They found that purchasers’ decisions are not rational because decisions did not depend on either the value or the duration of the project, although these variables were key determinants of project complexity. Soliño and Gago de Santos (2016) established a theoretical model based on transaction costs to compare the negotiation method and the open tendering in PPP. In their view, not all projects of higher complexity required the use of the negotiation method. The use of negotiation procedures is facilitated only in projects with a high degree of innovation and where important aspects of the quality of assets or services cannot be identified.

In addition, some literature has also focused on the impact of other factors. For example, in studies of foreign procurement practices, influencing factors include the level of competition (Bajari et al., 2009; Chong et al., 2014), electoral pressure (Chong et al., 2013), past performance and reputation of contractors (Bajari et al., 2009), and the capacity of the procurement officials (Chong et al., 2014). In the study of China’s procurement practices, influencing factors included the investment amount, duration, operation model, industry type, region, the provisions of laws and regulations, and the advice of advisory bodies (Wang et al., 2019).

To sum up, existing studies had laid a solid foundation for the selection of the procurement model, but there has been less previous evidence for explaining the preference for tendering method of PPP projects in China.

The selection of procurement models is a process of discretionary power exercised by procurement officials. Currently, China’s procurement system provides space for discretion in the selection of the negotiation method for complex projects, but the preference for the tendering method suggests that there may be other factors that limit this discretion. In China, different regulators have different preferences for procurement models because of the existence of two systems of procurement rules and regulatory authorities, and there is an incentive for authorities to use their regulatory power to demand preference for their rules (Li, 2015). Therefore, we assume that regulatory competition may lead to a preference for tendering method. Moreover, the *Government Procurement Law* and the *Tendering and Bidding Law* both prioritize the prevention of corruption and the value of transparency, and they provide for the preferred application status of the tendering method. Thus, the preference for procurement rules and the risk-averse behavior of officials may lead to the preference for the tendering method. In addition, the suppliers prefer to compete by tendering to avoid secondary tendering. Then the tacit interaction between suppliers and purchasers may have an inverse effect on the selection of procurement models. Consequently, this study focuses on the effects of the above factors.

To better observe these effects, we use the logit regression model to examine the influence of regulatory competition, risk aversion preference, and tacit interaction on procurement models selection based on the data of 8,926 PPP projects from 2009 to 2021 in China.

The academic contributions are as follows: through empirical study, we found that regulatory competition, risk aversion preference, and tacit interaction positively promote the preference for tendering in PPP procurement in China. This not only supplements the research on the influence factors but also provides explanatory evidence for the preference for tendering in China. For policymakers, we provide some advice for optimizing procurement of officials’ selection of procurement models along three dimensions: regulatory competition, risk aversion preferences, and tacit interaction. They can promote the realization of PPP procurement performance. In addition, for other countries promoting PPP in the international community, the analysis of China’s large-scale PPP project data can provide empirical evidence for their PPP project procurement.

The following arrangements of this study are as follows: The second part is the theoretical analysis. The third part shows the research design and data sources. The fourth part tells the empirical results. The fifth part makes the heterogeneity analysis. The sixth part is discussion. The seventh part is the conclusion and enlightenment.

## Theoretical Analysis

### Types and Features of the Procurement Model

In terms of the classification of procurement models, we refer to the classification in Chong et al. (2013); Soliño and Gago de Santos (2016), Baldi et al. (2016) and divide procurement models into tendering and negotiation. Nevertheless, different international organizations and countries put forward different expressions on the classification of procurement models. It leads to the different classification of tendering and negotiation methods in existing studies. Therefore, to classify tendering and negotiation more accurately, taking China’s procurement models as the reference, we compared and analyzed the types and characteristics of procurement models in the four major international procurement laws and obtained the following results:

First, tendering means that the purchaser invites non-specific (or specific) legal persons or other organizations to bid by tendering announcement (or tendering invitation). It aims to achieve three goals: (a) to maintain integrity, prevent corruption, and effectively solve the agency problem. (b) to achieve the economic benefits of government contracts. (c) to provide equal opportunities for all those who strive for government contracts (Dekel, 2007). Conversely, the disadvantage of tendering is that PPP contracts may be renegotiated more frequently in the future (Estache et al., 2009) and then increase procurement costs. In China, tendering is divided into open tendering and invited tenders. The difference between the two is whether to limit the number of suppliers. Similar procedures to open tendering include the Open procedures of the EU and the request for tendering of the World Bank (WB). Similar procedures to invited tendering include selective tendering of the World Trade Organization (WTO), the restricted procedure of the EU, and restricted tendering of the United Nations Commission on International Trade Law (UNCITRAL) (see Table 1). In PPP procurement in China, tendering includes open tendering and invited tenders.

**TABLE 1:** Comparison of types of procurement models.

Type	China (CN)	World Trade Organization (WTO)	European Union (EU)	United Nations Commission on Trade Law (UNCITRAL)	World Bank (WB)
Tendering	Open tendering	Open tendering	Open procedure	Open tendering	Request for Tender
	Invited tendering	Selective tendering	Restricted procedure	Restricted tendering	–
Negotiation	Competitive consultation	–	Competitive dialog	Request for proposals	Request for proposals
	Competitive negotiation	Restricted tendering	Competitive procedure with negotiation	Competitive negotiation	–
	Two-stage tendering	–	–	Two-stage tendering	–
Source	*Government Procurement Law*	*Government Procurement Agreement*	*Public Sector Procurement Directive (2014/24/EC)*	*Public Procurement Model Law*	*Investment Project Loan (IPF) Borrower Procurement Rules*

Second, negotiation means that the purchaser establishes a negotiation team to communicate with qualified suppliers on the procurement target. Suppliers submit response documents and quotations according to the requirements of negotiation documents, then the purchaser reviews and determines the concluded suppliers. The negotiation has multiple advantages: (a) it reduces the incompleteness of the contract, thus reducing the risk of renegotiation (Nunzia et al., 2016). (b) It saves the procurement period. The effectiveness of communication is a key factor to promote the effectiveness and efficiency of PPP. Frequent and effective communication between the public and private sectors helps to understand each other’s needs and avoid potential problems, thus reducing the procurement period (Liu et al., 2016). Third, it can promote innovation. Negotiation can tailor procurement contracts according to the needs of contracting agencies, and this is conducive to the innovation of PPP (Soliño and Gago de Santos, 2010). On the contrary, the limitations of the negotiation method are that it is less transparent and leaves more space for corruption (Baldi et al., 2016). In China, the negotiation method mainly includes competitive consultation, competitive negotiation, and two-stage tendering. The difference between competitive consultation and competitive negotiation is that the former chooses the best supplier for a comprehensive evaluation, while the latter chooses the lowest bidder. Similar procurement models to competitive consultation include the competitive dialog of the EU, the Request for Proposals of the UNCITRAL, and the WB. Similar procurement models to competitive negotiation include restricted tendering in the WTO and competitive procedures with negotiations in the EU. In addition, two-stage tendering could also be included in the category of negotiating method. It means that the tendering can be carried out in two stages when a project is technically complex, or the procurement specifications cannot be accurately formulated. In the first stage, procurement specifications shall be determined according to the technical suggestions submitted by the bidder, and tender documents shall be prepared. In the second stage, the bidder shall provide the best technical scheme and quotation according to the tender documents prepared. The UNCITRAL also regulates this method (see Table 1). In PPP procurement in China, negotiation includes competitive consultation and competitive negotiation.

### Analysis of the Influencing Factors of Procurement Model Selection

#### Regulatory Competition

In China, the Ministry of Finance (MoF) and the National Development and Reform Commission (NDRC) had made conflicting provisions for the selection of procurement models, respectively. This phenomenon can be discussed by the theory of regulatory competition. Regulatory competition means that different regulators gain competitive advantages by creating diverse laws to attract resources and mobile factors of production (Amit, 2010). That is, multiple regulatory systems are in competition with each other for the same regulatory matter. Under the theory of regulatory competition, there are multiple systems of rules, and the market can choose to apply one of them in its favor. At the same time, there is an incentive for the regulators to continually optimize their rules for the market to apply (Romano, 2005). A famous case is the global competition in the securities market. That is, there are multiple systems of stock exchange regulation around the world. Listed companies choose stock exchanges based on their interests and this creates competition between exchange rules. This regulatory competition is considered to be benign. Exchanges have an incentive to continuously optimize their rules to attract listed companies.

A regulatory overlap is a typical form of regulatory competition. It means that different agencies share the same regulatory authority in the same regulatory space (Li, 2015). It has many drawbacks, such as increased administrative costs, inefficient management, and reduced public trust in government (Li, 2015; Nay, 2021).

This phenomenon is prominent in PPP procurement in China. The procurement scale of PPP projects is huge, and the interests in the departments are also large. Moreover, the *Tendering and Bidding Law* and the *Government Procurement Law* give different departments the leading regulatory power. Therefore, the MoF and the NDRC have issued some regulations to regulate PPP respectively. Qin et al. (2021) point out that in the supervision of PPP projects in China, a dual-center management pattern has been formed between the MoF and the NDRC, and there is a competitive relationship between them.

At the level of PPP procurement model selection, regulation competition is reflected in the conflicts between the MoF and the NDRC. To be specific, on the one hand, the documents^6^ led by the MoF required that procurement model selection should be based on the characteristics of the project. When the project is complex, the purchaser should choose the negotiation method. On the other hand, the laws and regulations promulgated by the NDRC require that the tendering method must be selected under certain conditions. What’s more, the NDRC forces the market to choose its rules through administrative sanctions. For example, the documents^7^ issued by the NDRC required that all infrastructure PPP projects involving engineering and construction shall adopt the tendering method, otherwise they shall bear the legal responsibilities. We can see that the rules of the NDRC are more mandatory and binding than those of the MoF. This makes the selection of procurement models deviate from decision rationality and prefer the tendering method. To sum up, we propose:

Hypothesis 1: The regulatory competition affects the selection of procurement models. Under the influence of the MoF’s regulations, the application of negotiation has increased, especially in complex PPP projects. Under the influence of NDRC’s regulations, PPP projects will be more inclined to select tendering.

#### Risk Aversion Preference

The prevention of corruption is widely recognized as an important value of procurement law (Caillaud and Lambert-Mogiliansky, 2020; Xiao, 2020; Fazekas et al., 2021). It has influenced the design of procurement law, especially the traditional procurement law. For instance, transparency is considered to be an important principle of procurement law to support the value objective of corruption prevention (Neu et al., 2015). Similarly, the tendering method was given priority application status in traditional government procurement laws because it helps to prevent corruption and maximizes competition and transparency (Dekel, 2007), even if the excessive application of tendering may result in a loss of efficiency (Jiang, 2018). In addition, procurement law is designed to limit the discretion of procurement officials as much as possible for fear that they may be abused based on corrupt purposes (Gong, 2006). However, there is a potential risk of corruption in the application of the negotiation method (Baldi et al., 2016). As a result, traditional procurement law treats the negotiation method as an exceptional procurement model and imposes strict conditions on it and requires approval. This means that purchasers are subject to additional accountability when using the negotiation method. As can be seen, the selection of procurement models with a predominantly tendering method and an exception for negotiations is an important feature of traditional procurement law and is an important step to prevent corruption.

Correspondingly, in China, the *Government Procurement Law* has made the prevention of corruption a major value and has given priority to the tendering method. Likewise, considering the prevention of corruption, the *Tendering and Bidding Law* makes tendering the only procurement model and eliminates the negotiation method, which was widely available in previous tender and bidding rules and has been proposed in the *Tendering and Bidding Law (draft)* (Zeng, 1999). Thus, despite the discretion to select a more flexible procurement model based on complexity that remains in the existing rules, there is a conflict between this discretion and the primacy of the tendering method. It can leave procurement officials often facing confusion about the application of the law. The negative consequences of this confusion can be amplified by the risk-averse behavior of officials in situations, where there is a high demand for transparency or a tougher anti-corruption environment. Further, it leads to procurement officials procuring for process rather than for value (Cao, 2016).

To be specific, officials are self-interested in exercising their discretion, and the motives of safety and self-protection constantly drive them to seek a haven from the bureaucratic logic of doing things by the rules (Han, 2013). For procurement officials, open tendering is the least risky option. Among PPP procurement, open tendering is so popular because of its fairness and transparency. It can limit corruption and local political favoritism, thereby improving public sector accountability and overall welfare (Baldi et al., 2016). Meanwhile, because the value of corruption governance played an important role at the beginning of the legislation in China, open tendering was stipulated as the main procurement model (Xiao, 2020). Thus, even when projects are complex and lack competition, government officials can select open tendering to avoid suspicion of preferential treatment or corruption associated with negotiations (Chong et al., 2013). Given the above, we propose:

Hypothesis 2: The risk aversion preference makes PPP projects more likely to select tendering.

#### Tacit Interaction

In the theory of street-level bureaucracy, more and more scholars have paid attention to the influence of the interaction between grassroot officials and service objects on the exercise of discretion. Policy implementation is a process of interaction between grassroots officials and their service objects (Lotta and Marques, 2020). At present, there is a general “tacit interaction” between front-line administrators and audiences in China. Both parties in the interaction are clear about their motivations and orientation and take corresponding actions according to their mutual conditions and characteristics (Chen and Lu, 2013).

Interactions between purchasers and suppliers are more common in PPP projects. To be specific, in the process of determining procurement specifications, the project with complex technology should carry out the demand survey for the market^8^. Meanwhile, in PPP projects, the existing system provides the following paths for market participation in decision-making. First, the project can be initiated by the private sector, which will recommend potential PPP projects to the finance department through a project proposal^9^. The second is market testing, which is testing the market acceptance of government procurement conditions and market competition. Third, it is important to seek the suggestions of potential suppliers when preparing the implementation plan^10^.

Nevertheless, inappropriate interactions can easily lead to decisions that deviate from rationality in the selection of procurement models. Specifically, on the one hand, for purchasers, the generation of procurement decisions requires interaction with the private sector. In complex PPP projects, the government needs to communicate with the private to clearly and accurately define specifications. What is more, the purchaser needs to understand and consider the demand of private sectors through interaction to attract private investment. On the other hand, the private sectors, especially construction enterprises, all hope to participate in the competition through tendering. The reasons are as follows: according to the *Regulations on the Implementation of the Tendering and Bidding Law* of China, in general, after the franchise project investor is selected, the project construction should be subject to secondary tendering. Only the investors selected through tendering method can construct, produce or provide by themselves and may not conduct tendering anymore. Therefore, private sectors tend to participate in the competition through tendering to avoid secondary tendering and obtain the construction task. Then, the purchaser may deviate from its decision-making rationality to cater to the needs of private sectors and prefer the tendering method. To summarize, we propose:

Hypothesis 3: The tacit interaction makes PPP projects more likely to select tendering.

## Research Design and Data Sources

### Sample Data and Sources

From March 1, 2009 to March 20, 2021, the number of projects in the procurement and implementation stages was 9, 146. After removing central level projects, single-source procurement projects (156), and projects for which data were not available (60), 8, 926 projects remained. Among them, 7, 228 PPP projects were implemented through tendering and 1, 698 through negotiation.

Sample data sources are as follows: The data of PPP projects are partly from the Wind database, including project name, industry type, duration, investment, return model, demonstration type, region, etc. The data of procurement models come from the official website of the PPP Center of MoF, and the data acquisition method is manual sorting.

### Model

The dependent variable is the selection of procurement models. It is a dichotomous variable, so the Logit regression model is adopted (Chen, 2015). Tendering is 1 and negotiation is 0.

The independent variables are divided into regulatory competition, risk aversion preference, and tacit interaction. Specifically:

(a) In the aspect of regulatory competition, we mainly investigate the specific impact of the different regulations on procurement models selection between the MoF and the NDRC. First, we empirically tested the requirement of “complexity as the basis for procurement models selection” proposed by the MoF, and selected industry types as proxy variables of project complexity. The reason is that: Hart (2003) pointed out that the service measurability of infrastructure and public service projects was different. Compared with traditional infrastructure projects with relatively mature operational experience, non-traditional infrastructure projects are more complex (Tan et al., 2019). We used the classification method of NDRC to identify two categories of projects. Seven types of projects, including energy, transportation, water conservancy, environmental protection, agriculture, forestry, and municipal engineering, were labeled as traditional infrastructure projects, while other projects were labeled as non-traditional infrastructure projects. The above data were from the official website of the PPP Center of the MoF. Second, to investigate the influence of the documents of the MoF on the selection of negotiation, we took the issuance time of the *Caijin [2014] No. 113* as the node and divided the PPP projects into two groups, because the document requires that the negotiation method should be chosen for projects of high complexity. Third, to observe the influence of NDRC regulations on procurement model selection, we took the issuance time of the *Fagai Touzi [2016] No. 2231* as the node and divided the PPP projects into two groups, because the document specifies that all infrastructure PPP projects involving engineering and construction shall adopt the tendering method.
(b) As for the risk aversion preference of procurement officials, we chose local government transparency as a proxy variable. The more transparent the government is, the more likely corruption will be exposed, and the stronger the preference of officials to avoid risks. As is well-known, the corruption problem is difficult to expose because it has strong concealment and involves the corrupt party’s deliberate planning and hidden behavior. However, the disclosure of government information helps to weaken the degree of information asymmetry and makes the operation of power more transparent and open, and then it reduces the tendency of government corruption (Li, 2016). The government transparency data comes from the Chinese Government Transparency Index Report released by the Chinese Academy of Social Sciences from 2013 to 2020. Considering the discontinuous release of transparency data of provinces and the situation of cross-year release, we referred to the practice of Wang and Ma (2017) and used the mean of the 2015 and 2017 data to represent the 2016 data.
(c) On the measurement of tacit interaction, we used whether the winning bidder is the construction industry^11^ as a standard to examine its influence on the selection of procurement models. If the private union wins the bid, the leader’s industry type is mainly investigated. This is because the leader represents the overall interests of the union. The interaction between them and the procurement official is the most typical. The data of the winning bidder come from the official website of the PPP Center, and the data of industry type comes from Qichacha.

In addition, control variables include investment, regions, return models, operation models, project demonstration types, initiated time, and duration. The reasons are as follows: Reeves et al. (2015, 2017), Carter et al. (2019), Palcic et al. (2019) regarded investment as a proxy variable of project complexity, but Baldi et al. (2016) believed that there are limitations in the investment as a proxy variable of project complexity. Therefore, we took the investment as a control variable. Moreover, this study also controls for most of the characteristics of PPP projects such as regions, return models, operation models, project demonstration types, initiated time, and duration. Even so, there may be other factors related to the selection of procurement models, but we cannot include all possible factors due to data limitations. In this model, when observing the influence of an independent variable, other independent variables in the model are controlled.

The regression model is as follows:


(1) $$P\,M_{m}=\alpha_{m}+\beta_{m\,1}\,X_{i\,1}+\ldots +\beta_{m\,k}\,X_{i\,k}+\varepsilon_{m}$$

The independent variables are *X*_*i*1_⋯*X*_*ik*_ respectively. The coefficients are *β_*m1*_,…, β_*mk*_*, respectively. In addition, we performed the variance inflation factor (VIF) test to judge multicollinearity. We found that VIF values of all variables are less than 2. It proved that there is no serious multicollinearity problem in this model. The variable description is shown in Table 2.

**TABLE 2:** Description of variables.

Type	Variable				Description	Data source
Dependent variable	Procurement model				Dummy variables, negotiation = 0, tendering = 1	Official website of the PPP Center of the MoF
Independent variables	Regulatory competition	MOF’s regulation	Project complexity	Industry type	Dummy variable, infrastructure project = 1, public service project = 0	Wind Database
			issuance of *Caijin [2014]NO. 113*		Dummy variable, PPP projects initiated before November 29, 2014 = 0, PPP projects initiated after November 29, 2014 = 1	
		NDRC’s regulation	issuance of *Fagai Touzi[2016]NO. 2231*		Dummy variable, PPP projects initiated before October 24, 2016 = 0, PPP projects initiated after October 24, 2016 = 1	
	Risk aversion preference		Government transparency		Continuous variable, government Transparency index by province.	Report of Chinese Government Transparency Index (2013–2020)
	Tacit Interaction		Whether the winning bidder is a construction enterprise		Dummy variable, the winning bidder is non-construction enterprise = 0, the winning bidder is construction enterprise = 1.	Qichacha
Control variables	Investment				Continuous variable, PPP project investment amount, logarithm	Wind Database
	Duration				Continuous variable, cooperation period of PPP	
	Region				Multi-valued variable, eastern region = 1, central region = 2, western region = 3	
	Return model				Multi-valued variable, government-pay = 1, viability gap funding = 2, user-pay = 3	
	Operation model				Multi-valued variable, BOT = 1, BOO = 2, TOT = 3, ROT = 4, MC = 5, OM = 6, Others = 7, TOT+BOT = 8, TOT+BOO = 9	
	Demonstration type				Multi-valued variable, national demonstration project = 1, provincial demonstration project = 2, municipal demonstration project = 3, others = 4	
	Initiated time				Multi-valued variable, 2009 = 1, 2010 = 2, 2011 = 3, 2012 = 4, 2013 = 5, 2014 = 6, 2015 = 7, 2016 = 8, 2017 = 9, 2018 = 10, 2019 = 11, 2020 = 12, 2021 = 13	

## Empirical Results

### Descriptive Statistics

**FIGURE 2:**
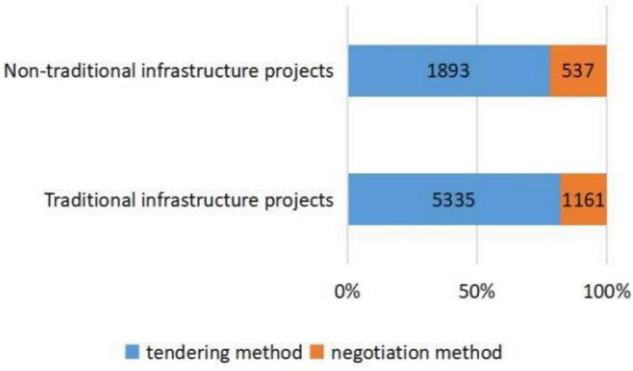
Distribution of procurement models selection for different industry types.

**FIGURE 3:**
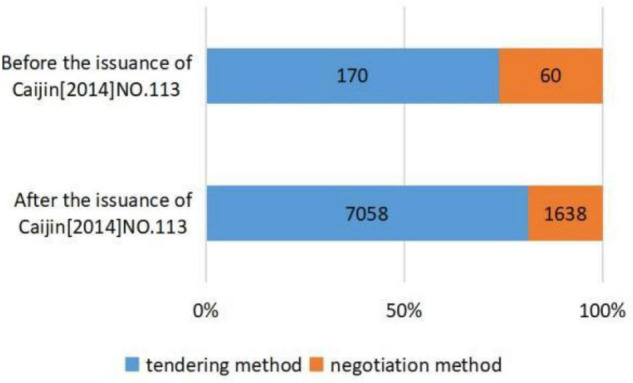
Distribution of procurement models selection before and after the issuance of *Caijin [2014]NO. 113.*

**FIGURE 4:**
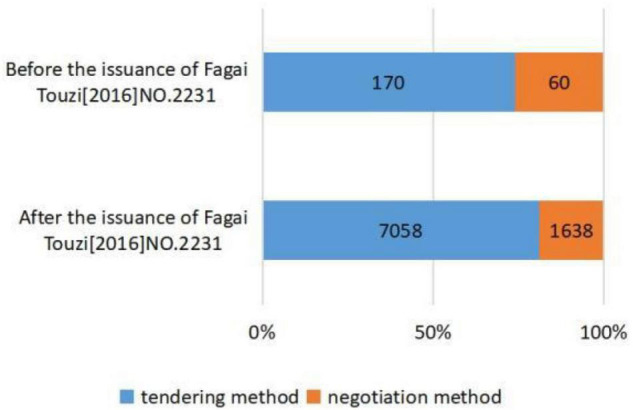
Distribution of procurement models selection before and after the issuance of *Fagai Touzi[2016]NO. 2231.*

**FIGURE 5:**
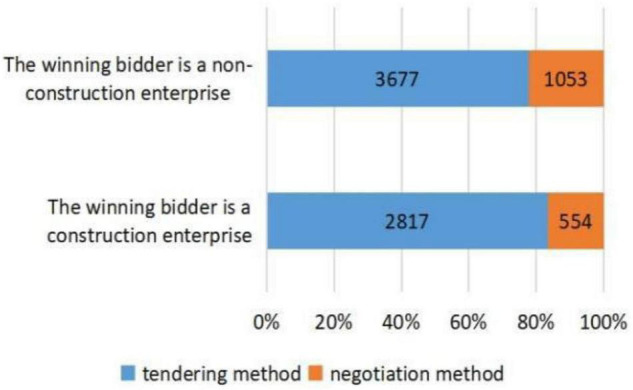
Distribution of procurement model selection under different types of winning bidders.

Figures 2–5, respectively, presents the distribution of procurement model selection for different industry types, different initiated times, and different enterprise types of winning bidders. Figure 2 shows that traditional infrastructure projects adopt more tendering and have a higher proportion compared with non-traditional infrastructure projects. Figure 3 illustrates that compared with PPP projects initiated before the issuance of *Caijin [2014]NO. 113*, the projects initiated after the issuance of *Caijin [2014]NO. 113* adopted more negotiation. Figure 4 shows that compared with PPP projects initiated before the issuance of *Fagai Touzi[2016]NO. 2231*, the projects initiated after the issuance of *Fagai Touzi[2016]NO. 2231* adopted more tendering and had a higher proportion. Figure 5 indicates that the proportion of PPP projects won by construction enterprises adopting tendering is relatively high. Table 3 demonstrates the descriptive statistics of the transparency index of PPP projects in provinces corresponding to the two groups of procurement models. Admittedly, descriptive statistics is only a preliminary description of variables, and the accurate relationship between explanatory variables and explained variables needs to be carried out through normative regression.

**TABLE 3:** Descriptive statistics of transparency in provinces where PPP projects are located under different procurement models (Unit: none).

Type	Mean	Min	p25	p50	p75	Max
Negotiation	69.96686	39.03	64.17	71.97	77.07	85.91
Tendering	69.95785	37.7	65.03	71.2	76.26	88.61
Total	69.95956	37.7	65.03	71.27	76.26	88.61

### Regression Results

Table 4 shows the results of logit regression on equation (1). To facilitate the interpretation of regression results, odds ratios rather than coefficients are reported. As can be seen:

(a) at the level of regulatory competition, under the control of other variables: first, compared with non-traditional infrastructure projects, the probability of tendering for traditional infrastructure projects increased significantly by 45.7%. The results verify the influence of project complexity on procurement model selection. Second, after the issuance of *Caijin [2014]NO. 113*, the probability of tendering is significantly reduced by 73.7%. Third, after the issuance of *Fagai Touzi[2016]NO. 2231*, the probability of tendering increased significantly by 46.1%. In summary, it can be seen that the requirement of project complexity as the basis of procurement model selection proposed by the MoF has been applied in practice. The simpler the project is, the more likely it is to select tendering. Meanwhile, the issuance of *Caijin [2014]NO. 113* has significantly inhibited the application of tendering. Instead, the release of the *Fagai Touzi[2016]NO. 2231* significantly promoted the application of tendering. Thus, hypothesis 1 is verified.
(b) As for risk aversion preference, when controlling for other variables, with each unit increase in the local government transparency index, the probability of selecting tendering increases by 2.5%. Therefore, the region with a stronger risk aversion preference tends to select tendering. Hypothesis 2 is verified.
(c) In terms of tacit interaction, compared with PPP projects won by non-construction enterprises, PPP projects won by construction enterprises have a 56.3% higher probability of selecting tendering under the condition of controlling other variables. This indicates that the interaction between construction enterprises and procurement officials makes PPP projects more likely to select tendering. Hypothesis 3 is verified.

**TABLE 4:** Baseline regression result.

Variables	Odds Ratio	Std. Err.
traditional Infrastructure projects	1.457***	(0.0997)
PPP projects initiated after the issuance of *Caijin [2014]NO.113*	0.263***	(0.0555)
PPP projects initiated after the issuance of *Fagai Touzi[2016]NO.2231*	1.461***	(0.148)
Government transparency	1.025***	(0.00352)
The winning bidder is a construction enterprise	1.563***	(0.102)
Duration	0.998	(0.00461)
Investment(log)	1.158***	(0.0280)
Region	0.983	(0.0358)
Return model	1.061	(0.0594)
Operation model	0.970**	(0.0125)
Demonstration type	1.124***	(0.0328)
Initiated year	1.580***	(0.0720)
Constant	0.00428***	(0.00208)
Observations	8,078	

Robust standard errors in parentheses ***p < 0.01, **p < 0.05.

### Robustness Test

#### Robustness of Key Variables

To test the robustness of key variables, we took the following two measures:

First, referring to the practice of Wang et al. (2019), we used the quantile regression method for government transparency. When both quantile regression results and baseline regression results are significant, it can be said that the relationship between the explanatory variable and explained variable is significant or robust. The quantile regression result illustrates that, compared with the regions with a transparency index lower than 64.3, the probability of selecting a tendering in the regions with a transparency index between 64.3 and 70.83 increases by 23.5%, the probability of selecting a tendering in the regions with a transparency index of 76.75–88.61 increases by 47.9%, the probability of selecting a tendering in the regions with transparency index between 76.75 and 88.61 increases by 97.3% (see Table 5). This result shows that with the increase of the transparency index, the probability of tendering selection is higher. It confirms the robustness of the baseline regression result.

**TABLE 5:** Quantile regression result.

Variables	Odds Ratio	Std. Err.
traditional Infrastructure projects	1.470***	(0.101)
PPP projects initiated after the issuance of *Caijin [2014]NO.113*	0.250***	(0.0533)
PPP projects initiated after the issuance of *Fagai Touzi[2016]NO.2231*	1.433***	(0.143)
Government transparency 2 (64.3–70.83)	1.235**	(0.111)
Government transparency 3 (70.83–76.75)	1.479***	(0.125)
Government transparency 4 (76.75–88.61)	1.973***	(0.176)
The winning bidder is a construction enterprise	1.579***	(0.104)
Duration	0.998	(0.00461)
Investment(log)	1.156***	(0.0279)
Region	0.953	(0.0356)
Return model	1.072	(0.0602)
Operation model	0.970**	(0.0125)
Demonstration type	1.124***	(0.0328)
Initiated year	1.633***	(0.0779)
Constant	0.0146***	(0.00635)
Observations	8,078	

Robust standard errors in parentheses ***p < 0.01, **p < 0.05.

Second, we replaced the proxy variables and run regressions. The degree of corrupt governance is used as a proxy variable for risk aversion preferences instead of the government transparency index. Procurement officials’ risk aversion preferences are closely related to the governance risks they face. Based on the importance of the value of corruption prevention in the procurement rules (Xiao, 2020), the governance risk faced by procurement officials mainly stems from the accountability for their corrupt behavior. The higher the degree of corruption governance in a region is, the stronger the risk aversion preferences of procurement officials. As for corruption governance, Fisman and Gatti (2002) used the number of civil servants punished for abusing power as a measure of the corruption degree of each state in the United States. They pointed out that the civil servants’ abuse of power is a reasonable indicator to measure the level of corruption. Zhou and Tao (2009) adopted the total number of regional corruption cases as a proxy variable of the degree of corruption. Wang and Ma (2017) adopted the number of corruption and malfeasance crimes registered by the procuratorates in each region as the proxy variable. Based on the above research, we use the number of indictments for corruption and malfeasance crimes filed by the procuratorate as a proxy variable of the degree of corruption governance. The data were obtained from the PKULAW database. The regression result is shown in Table 6. We can see that as the number of indictments increases by one unit, the probability of tendering for PPP projects in the region increases by 41.4%. This further illustrates the promotion effect of risk aversion preference on the selection of tendering method.

**TABLE 6:** Regression result of substitute proxy variables.

Variables	Odds Ratio	Std. Err.
traditional Infrastructure projects	1.494***	(0.102)
PPP projects initiated after the issuance of *Caijin [2014]NO.113*	0.295***	(0.0591)
PPP projects initiated after the issuance of *Fagai Touzi[2016]NO.2231*	1.303***	(0.126)
Number of corruption and malfeasance indictments(log)	1.414***	(0.0822)
The winning bidder is a construction enterprise	1.541***	(0.101)
Duration	0.998	(0.00461)
Investment(log)	1.164***	(0.0280)
Region	0.971	(0.0345)
Return model	1.086	(0.0607)
Operation model	0.970**	(0.0125)
Demonstration type	1.116***	(0.0326)
Initiated year	1.528***	(0.0642)
Constant	0.00164***	(0.00100)
Observations	8,101	

Robust standard errors in parentheses ***p < 0.01, **p < 0.05.

#### Robustness of Estimation Method

To test the robustness of the estimation method, we replaced the Logit regression with Probit regression. We can find that the result from Table 7 is consistent with the conclusions obtained from the baseline regression. This illustrates that the regression method adopted in this study is robust.

**TABLE 7:** Probit regression result.

Variables	Odds Ratio	Std. Err.
traditional Infrastructure projects	1.237***	(0.0483)
PPP projects initiated after the issuance of *Caijin [2014]NO.113*	0.473***	(0.0561)
PPP projects initiated after the issuance of *Fagai Touzi[2016]NO.2231*	1.256***	(0.0715)
Government transparency	1.014***	(0.00200)
The winning bidder is a construction enterprise	1.291***	(0.0478)
Duration	0.999	(0.00264)
Investment(log)	1.091***	(0.0149)
Region	0.987	(0.0205)
Return model	1.031	(0.0329)
Operation model	0.982**	(0.00727)
Demonstration type	1.073***	(0.0184)
Initiated year	1.287***	(0.0321)
Constant	0.0473***	(0.0130)
Observations	8,078	

Robust see form in parentheses ***p < 0.01, **p < 0.05.

## Heterogeneity Analysis

Through the above analysis, we found that regulatory competition affects the selection of procurement models, while risk aversion preference and tacit interaction significantly promote the application of tendering. To further prove this logic, we conducted heterogeneity analysis from two aspects: regions and return models.

### Heterogeneity of Regions

According to different city sizes, we divided the cities launching PPP projects into provincial capital cities and municipalities, and ordinary cities. Table 8 shows the result. As can be seen:

(a) in terms of regulatory competition, the regulations of the MoF and the NDRC have opposite effects on the selection of procurement models. This is similar to the baseline regression result. What’s more, we also find that the above factors have a higher influence on provincial capitals and municipalities. This is due to provincial capitals and municipalities having a higher level of policy enforcement than ordinary cities. Consequently, they are more susceptible to regulatory competition.
(b) As for risk aversion preference, higher government transparency significantly promoted the application of tendering. It is consistent with the baseline regression result. In addition, we find that the impact of risk aversion preference on provincial capitals and municipalities is higher. This is because the higher the level of a city is, the easier it is to be held accountable (Simonsen et al., 2001). As a result, procurement officials in higher-level cities are more susceptible to risk aversion. This result further confirms the analysis of risk aversion preference in this study.
(c) At the level of tacit interaction, in line with the baseline regression result, tendering applications increase significantly in these projects, where the winning bidder is a construction enterprise. Furthermore, we find that tacit interaction has a higher influence on provincial capitals and municipalities. This is owing to large-scale cities generally having strong organizational capacity, and they can provide a more favorable environment for PPP (Tan et al., 2019), thus, attracting more private sectors to participate in the interaction. This further supports our analysis of tacit interaction.

**TABLE 8:** Regression result of regional heterogeneity analysis.

Variables	Provincial Capital Cities and Municipalities	Ordinary Cities
Traditional Infrastructure projects	1.454* (0.321)	1.489*** (0.110)
PPP projects initiated after the issuance of *Caijin [2014]NO.113*	0.186*** (0.109)	0.293*** (0.0679)
PPP projects initiated after the issuance of *Fagai Touzi[2016]NO.2231*	2.620*** (0.772)	1.375*** (0.157)
Government transparency	1.062*** (0.0101)	1.020*** (0.00390)
The winning bidder is a construction enterprise	2.053*** (0.383)	1.468*** (0.106)
Duration	Controlled	Controlled
Investment(log)	Controlled	Controlled
Region	Controlled	Controlled
Return model	Controlled	Controlled
Operation model	Controlled	Controlled
Demonstration type	Controlled	Controlled
Initiated year	Controlled	Controlled
Constant	0.00195*** (0.00286)	0.00671*** (0.00373)
Observations	1,063	6,260

Robust see form in parentheses ***p < 0.01, *p < 0.1. Due to space limitation, regression results of control variables are not reported in this table and are kept for reference, the same as below.

### Heterogeneity of Return Models

The return models of PPP projects include government-pay, viability gap funding, and user-pay. Table 9 shows the regression result. As can be seen:

(a) as for regulatory competition, in the government-pay and viability gap funding projects, the regulations of the MoF and the NDRC have a reverse impact on the procurement model selection, and it is in line with the baseline regression result. Conversely, this effect is not significant in user-pay projects. Moreover, by observing the degree of influence, it indicates that regulatory competition has the highest influence on government-pay projects. This is owing to the promotion of projects based entirely on government payments is restricted by strict regulations in China for reducing the pressure on local government spending and preventing the risk of local debt. Both the MoF and the NDRC called for the prudent promotion of government-pay projects and encouraged user-pay projects. Instead, user-pay projects that do not involve financial outlays are less regulated^12^. The higher degree of regulation makes procurement officers more likely to follow the rules when carrying out these projects with fiscal expenditure. As a result, they are more susceptible to regulatory competition.
(b) In terms of risk aversion preference, higher government transparency significantly promoted the application of tendering in government-pay and viability gap funding projects, and it is in keeping with the baseline regression result. Conversely, in user-pay projects, this effect is significantly inhibited. The reason is that: different return models pose different risks for procurement officials. Government-pay and viability gap funding projects have higher requirements and risks for the decision-making of procurement officials and higher because they involve financial expenditure. Therefore, procurement officials are more affected by risk aversion preference in government-pay and viability gap funding projects. This further confirms the analysis of risk aversion preference in this study. On the contrary, user-pay projects do not involve financial expenditure, and procurement officials have low decision-making risks. They are more likely to choose procurement models scientifically according to the characteristics of the project, rather than blindly favoring tendering method. Also, as transparency increases and the decision-making environment is optimized, this tendency becomes more pronounced.
(c) Tacit interaction significantly promoted the application of tender, and it is in accordance with the baseline regression result. Meanwhile, tacit interaction has the highest impact on user-pay projects. This is due to user-pay projects being the most market-oriented, and there is also more interaction between the purchaser and potential suppliers. Therefore, they are more susceptible to this factor. This further confirms the analysis of tacit interaction.

**TABLE 9:** Regression result of heterogeneity analysis of return models.

Variables	Government-Pay	Viability Gap Funding	User-Pay
Traditional Infrastructure projects	1.643*** (0.187)	1.445*** (0.133)	1.041 (0.265)
PPP projects initiated after the issuance of *Caijin [2014]NO.113*	0.129*** (0.0553)	0.338*** (0.0988)	0.628 (0.343)
PPP projects initiated after the issuance of *Fagai Touzi[2016]NO.2231*	1.537*** (0.235)	1.358** (0.195)	1.724 (0.761)
Government transparency	1.029*** (0.00543)	1.030*** (0.00498)	0.972* (0.0148)
The winning bidder is a construction enterprise	1.276** (0.127)	1.804*** (0.168)	2.128** (0.658)
Duration	Controlled	Controlled	Controlled
Investment(log)	Controlled	Controlled	Controlled
Region	Controlled	Controlled	Controlled
Return model	Controlled	Controlled	Controlled
Operation model	Controlled	Controlled	Controlled
Demonstration type	Controlled	Controlled	Controlled
Initiated year	Controlled	Controlled	Controlled
Constant	0.00557*** (0.00465)	0.00231*** (0.00156)	1.406 (2.493)
Observations	3,054	4,586	438

Robust see form in parentheses ***p < 0.01, **p < 0.05, *p < 0.1.

## Discussion

Through empirical research, we find that:

(a) the existence of regulatory competition between the MoF and the NDRC creates a dilemma for procurement model selection. Specifically, the requirement of “project complexity as the basis of procurement model selection” proposed by the MoF has been applied in practice. The more complex the project is, the more likely it is to select negotiation. This also proves the viewpoint of Carbonara et al. (2016) through empirical data. Meanwhile, the issuance of *Caijin [2014] No. 113* has significantly inhibited the application of tendering. Conversely, the issuance of *Fagai Touzi[2016]NO. 2231* has significantly promoted the application of tendering. This suggests that the existence of regulatory competition between the MoF and the NDRC has an opposite impact on the selection of procurement models.
(b) Risk aversion preference significantly promotes the application of tendering. This result confirmed Chong et al.’s (2013) view that government officials prefer to choose open tendering to avoid being suspected of preferential treatment or corruption related to negotiations through empirical data. This finding is consistent with our theoretical analysis. The reasons for this phenomenon are as follows: on the one hand, the existence of regulatory competition gives procurement officials discretion in the selection of procurement models. On the other hand, the high emphasis on the anti-corruption value at the beginning of government procurement legislation has affected the regulations of procurement model selection and gives priority to open tender. Therefore, the purchaser prefers tendering.
(c) Tacit interaction significantly promoted the application of tendering. Taking PPP procurement as the sample, this finding confirms the view that the interaction between grass-roots officials and service objects affects the exercise of officials’ discretion in street bureaucracy theory. This is owing to potential suppliers tending to participate in PPP projects through tendering to avoid secondary tendering, while purchasers tend to select tendering based on risk aversion preference. The common needs of both parties strengthen procurement officials’ selection of tendering.

However, whether these influences are conducive to the scientific selection of procurement models needs further discussion.

First, as for regulatory competition, although in the theory of regulatory competition, the supervision of multiple regulatory agencies on the same matter is helpful to avoid regulatory failure and bring more effective regulatory services (Kane, 1997, 1999), the existence of regulatory competition affects the orderly promotion of PPP procurement in China. Different PPP policymakers lead to different policy positions, strong interest conflicts, difficult coordination, and other problems. These problems make PPP practice unable to form stable expectations (Yu, 2016; Wang et al., 2021). Especially in the selection of procurement models, the existence of regulatory competition between the MoF and the NDRC leads to the selection dilemma of procurement officials in the exercise of discretion, and then results in conflicting consequences for the selection, finally affecting the scientific decision.

Second, procurement officials’ preference for avoiding risks exists objectively, but the institutional design of procurement model selection based on the value of corruption governance ignores the pursuit of procurement effect. Although the value of corruption governance occupies an important position in procurement legislation, with the continuous development and improvement of the function of procurement law, the value of internationally recognized procurement law has gradually changed from focusing on procedural regulations to result-oriented (Sue and Cao, 2016). In China, with the promotion of comprehensive budget performance management and the development of modern finance, the value of government procurement has gradually changed from procedural norms to performance-oriented (Jiang, 2014). That is, while preventing corruption, more attention needs to be paid to the procurement effect and quality (Liu, 2017). Therefore, the system of procurement model selection formed under the governance value of corruption should be re-examined, and the preference of procurement model selection caused by the risk aversion preference formed under this value should also be corrected. Furthermore, the tendering method is not a panacea. As noted in the introduction, the effectiveness of tendering has been questioned when facing complex procurement projects. Thus, the design of a tendering-oriented procurement method selection system should be optimized.

Third, in terms of tacit interaction, for complex PPP projects, purchasers need to interact with potential suppliers to clarify procurement specifications. Nevertheless, procurement law does not have the rationality and legitimacy for suppliers to influence the selection of procurement models and taking the demand of suppliers as the consideration standard is not in line with the requirements of procurement models selection. The interaction before the procurement process begins affects the scientific exercise of the discretionary power of procurement officials. Therefore, we must control for inappropriate interactions, otherwise, the value of fair competition in the procurement legal system will be undermined.

To sum up, regulatory competition, risk aversion preference, and tacit interaction all affect the scientific selection of procurement models.

Theoretical analysis shows that tendering and negotiation belong to different types of procurement. They have advantages and disadvantages respectively and apply to different situations. From the perspective of international experiences, appropriate procurement models are selected according to the characteristics of projects. If the improper influences of regulatory competition, risk aversion preference, and tacit interaction make procurement officials fail to select appropriate procurement models, it may lead to the failure to select optimal private sector, and thus make it difficult to achieve the procurement performance of PPP.

So, how do we optimize the selection of procurement models? Combined with our empirical research, we can focus on the following three aspects. To be specific:

(a) Reducing the adverse impact of regulatory competition on the procurement model selection. First, the important role of project complexity should be taken seriously in procurement model selection. It not only accords with the judgment of theoretical research but also accords with the regulations of the four international procurement laws. Take the newly revised United Nations legislation as an example, both the *UNCITRAL Model Law on Public Procurement* and the *UNCITRAL Model Law on Public-Private Partnerships* pointed out that when the project is complex and the procurement specifications are difficult to clearly define, the negotiation method should be selected^13^. Second, policymakers should unify PPP legislation, in particular, the selection of procurement models needs to be regulated uniformly. Third, the regulatory agency for PPP projects should be clarified and unified. A special agency for PPP projects at the central level, independent of the MoF and the NDRC, could be set up to break the fragmented supervision of different departments.
(b) Reducing the adverse impact of risk aversion preferences on the selection of procurement models. We should optimize the officials’ risk perception and help them to form a better preference for avoiding risks. On the one hand, we should take the realization of procurement performance as the main goal in the selection of procurement model and pay attention to the realization of efficiency value and corruption prevention. For this purpose, we can learn from the Provisions of the UNCITRAL Model Law on Public Procurement to further refine procurement models, standardize the negotiation process, optimize competition, and enhance transparency. It can not only achieve procurement performance but also achieve the goal of combating corruption and preventing the abuse of procurement models. On the other hand, we should place different procurement models on equal status to eliminate the dominance of open tendering.
(c) Reducing the adverse impact of tacit interactions on the selection of procurement models. The existing procurement system has provided a more standardized channel for bilateral communication. Therefore, when facing complex projects that require mutual interaction, the negotiation method should be selected. The purchaser shall interact with the supplier during the procurement process as prescribed by laws and regulations.

## Conclusion and Enlightenment

Under the background of the comprehensive implementation of budget performance management, it has become an important topic to promote the realization of PPP procurement performance, and the selection of procurement models plays an important role in the realization of procurement performance. At present, how to select the procurement model scientifically has attracted more and more attention from practical and academic circles, but the empirical research on the influencing factors of procurement model selection is still insufficient. From theoretical analyses, domestic and foreign procurement laws, and international PPP procurement practices, we can see that negotiation is more suitable for complex PPP projects. However, tendering is widely used in PPP projects in China. To explain the above phenomenon, we took 8,926 PPP projects from 2009 to 2021 in China as samples and empirically analyzed the influencing factors of procurement model preference for tendering by Logit regression. It is found that regulatory competition leads to the confusion of procurement model selection, while risk aversion preference and tacit interaction significantly promote the application of tendering. In addition, the heterogeneity analyses of regions and return models indicate that provincial capitals and municipalities are more susceptible to regulatory competition, risk aversion preference, and tacit interaction than ordinary cities. Compared with user-pay projects, government-pay and viability gap funding projects are more easily affected by regulatory competition and risk aversion preference and less affected by tacit interaction. These findings further confirm the influence of regulatory competition, risk aversion preference, and tacit interaction on procurement model selection.

The above findings bring policy implications for optimizing the selection of procurement models. It is no good or bad between tendering and negotiation, but if the improper influence of regulatory competition, risk aversion preference, and tacit interaction make procurement officials fail to select appropriate procurement models, it may lead to the failure of selecting optimal private sector, and then lead to the failure of PPP projects. Therefore, policymakers should:

(a) Reduce the influence of regulatory competition on the selection of procurement models from the following three aspects: attaching importance to the role of project complexity of procurement model selection, unifying the PPP legislation, and clarifying and unifying the regulatory agency.
(b) Reduce the impact on risk aversion preference for the selection of procurement models through the following two aspects: taking the realization of procurement performance as the main goal in the selection of procurement model, placing different procurement models on equal status to eliminate the dominance of open tendering.
(c) Reduce the influence of interaction between procurement officers and potential suppliers before the procurement process begins. When facing complex projects, procurement is conducted through a standardized negotiation process.

Whereas our analysis sheds light on questions such as why tendering method is preferred in China’s PPP projects. We do not analyze the effects of different procurement models through empirical evidence. What’s more, our research does not reveal whether the negotiation method reduces the likelihood of renegotiation in China’s practice. Analysis along these lines provides a potentially fruitful line of inquiry that can build on the initial contribution presented in this paper.

## Data Availability Statement

The raw data supporting the conclusions of this article will be made available by the authors, without undue reservation.

## Author Contributions

FGC contributed to the conception and design of the study, made a substantial contribution to the introduction, theoretical analysis, discussion, and conclusions including but not limited to substantive revision of the parts thereof, and guided the literature review. CW organized the database, performed the statistical analysis, and wrote the first draft of the manuscript. In addition, both authors contributed to manuscript revision, read and approved the submitted version, and also agreed to be accountable for all aspects of the work in ensuring that questions related to the accuracy or integrity of any part of the work are appropriately investigated and resolved.

## Conflict of Interest

The authors declare that the research was conducted in the absence of any commercial or financial relationships that could be construed as a potential conflict of interest.

## Publisher’s Note

All claims expressed in this article are solely those of the authors and do not necessarily represent those of their affiliated organizations, or those of the publisher, the editors and the reviewers. Any product that may be evaluated in this article, or claim that may be made by its manufacturer, is not guaranteed or endorsed by the publisher.

## Footnotes

1 See Section “Types and Features of the Procurement Model”.

2 See Article 54 of the *Public Procurement Law* (Draft for Comments).

3 See Article 30 of the *Government Procurement Law*.

4 See Article 13 of the *UNCITRAL Model Law on Public-Private Partnerships*.

5 Note: Statistical results are subject to the data in the PPP Central project database of the Ministry of Finance, and there are no two-stage tendering projects in the statistics. In addition, although single source procurement includes negotiation process, it is not included in the negotiation methods discussed in this paper because of the particular circumstances in which it is applied.

6 See article 11 of the *Notice on Printing and Distributing The Operating Guide for The Model of Public Private Partnership (Caijin [2014] No. 113)*.

7 See the *Notice on Printing and Distributing the Guidelines for the Implementation of Public-Private Partnership Projects in the Field of Traditional Infrastructure (Fagai Touzi[2016]NO. 2231)*.

8 See Article 11 of *the Notice of the MoF on the Issuance of Measures for the Management of Government Procurement Requirements (Cai Ku [2021] No. 22)*.

9 See Article 6 of the *Caijin [2014] No. 113.*

10 See Article 9 of the *Fagai Touzi[2016]NO. 2231.*

11 Construction enterprises include housing construction, building and installation industry, construction industry, building decoration, decoration and other construction industry, civil engineering construction industry.

12 See the *Guidance of the NDRC on Encouraging Private sectors to Participate in Public-Private Partnership (PPP) Projects (Fagai Touzi [2017] No. 2059)*, and the *Notice of the General Office of the MoF on Standardizing the Management of Project Database of public-Private Partnership (PPP) Comprehensive Information Platform (Caibanjin [2017] No. 92)*.

13 See article 13 of the *UNCITRAL Model Law on Public-Private Partnerships* and chapter II of the *UNCITRAL Model Law on Public Procurement*.
